# Acyl-CoA synthetase long-chain 5 genotype is associated with body composition changes in response to lifestyle interventions in postmenopausal women with overweight and obesity: a genetic association study on cohorts Montréal-Ottawa New Emerging Team, and Complications Associated with Obesity

**DOI:** 10.1186/s12881-016-0320-4

**Published:** 2016-08-11

**Authors:** Abishankari Rajkumar, Gilles Lamothe, Pierrette Bolongo, Mary-Ellen Harper, Kristi Adamo, Éric Doucet, Remi Rabasa-Lhoret, Denis Prud’homme, Frédérique Tesson

**Affiliations:** 1 Department of Biochemistry, Microbiology and Immunology, University of Ottawa, Ottawa, ON Canada; 2 Department of Mathematics and Statistics, University of Ottawa, Ottawa, ON Canada; 3 Interdisciplinary School of Health Sciences, University of Ottawa, Ottawa, ON Canada; 4 CHEO Research Institute, Ottawa, ON Canada; 5 Department of Pediatrics, University of Ottawa, Ottawa, ON Canada; 6 School of Human Kinetics, University of Ottawa, Ottawa, ON Canada; 7 Départment de Nutrition, Université de Montréal, Montreal, QC Canada; 8 Institut de Recherches Cliniques de Montréal, Montréal, QC Canada; 9 Institut de recherche de l’Hôpital Montfort, Hôpital Montfort, Ottawa, ON Canada; 10 Faculty of Health Sciences, 451 Smyth, Ottawa, ON K1H 8M5 Canada

**Keywords:** Genotype, Obesity, Lifestyle modifications, Fat metabolism

## Abstract

**Background:**

Genetic studies on Acyl-CoA Synthetase Long-Chain 5 (ACSL5) demonstrate an association between rs2419621 genotype and rate of weight loss in women with obesity in response to caloric restriction. Our objectives were to (1) confirm results in two different populations of women with overweight and obesity (2) study rs2419621’s influence on body composition parameters of women with overweight and obesity following lifestyle interventions.

**Methods:**

rs2419621 genotype was determined in women with overweight and obesity who participated in the Montréal-Ottawa New Emerging Team (MONET *n* = 137) and Complications Associated with Obesity (CAO *n* = 37) studies. Genotyping was done using TaqMan MGB probe-based assay. Multiple linear regression analyses were used to test for associations.

**Results:**

When studying women with overweight and obesity, rs2419621 [T] allele carriers had a significantly greater decrease in visceral fat, absolute and percent fat mass and a greater increase in percent lean mass in response to lifestyle intervention in comparison to non-carriers. Studying only individuals with obesity showed similar results with rs2419621 [T] allele carriers also displaying a significantly greater decrease in body mass index following the lifestyle intervention in comparison to non-carriers.

**Conclusion:**

Women with overweight and obesity carrying the ACSL5 rs2419621 [T] allele are more responsive to lifestyle interventions in comparison to non-carriers. Conducting such genetic association studies can aid in individualized treatments/interventions catered towards an individual’s genotype.

## Background

Obesity, recently defined as a disease by the American Medical Association, and overweight are major risk factors for a variety of chronic diseases including cardiovascular diseases, type 2 diabetes and cancer [1]. Genetic and epigenetic mechanisms, mediated by lifestyle and environmental exposures have been implicated in the development of obesity and these chronic diseases [2, 3].

As a critical component of metabolic pathways, fatty acyl-CoA molecules are known to be implicated in energy production by β-oxidation, energy storage through lipid biosynthesis and as lipid components of the cell. The acyl-CoA synthetases long-chain (ACSL) stimulates intracellular free long-chain fatty acids by converting them to fatty acyl-CoA molecules. Members of the ACSL family, ACSL 1, 3, 4, 5 and 6 are characterized by varying subcellular localization, fatty acid substrate and tissue specificity [4]. ACSL5 is present in various species including humans and rodents, while also being distributed in a wide range of tissues including skeletal muscle, liver, and brain [5]. ACSL5 has been detected in rat liver cytosol, endoplasmic reticulum and mitochondrial outer membrane [6]. Increased ACSL5 protein levels have been observed during food deprivation in rats [6]. Furthermore, ACSL5 plays a role in facilitating fatty acid channelling between anabolic lipid synthesis and catabolic β-oxidation pathway [6, 7]. Previous research conducted in our laboratory demonstrated that among 8 polymorphisms along the ACSL5 gene, only the common single nucleotide polymorphism (SNP) rs2419621, found in the promoter region, displayed a significant association with rate of weight loss response in women with obesity participating in a weight management program (which included an initial 6-week period of 900 kcal meal replacement) [8]. Characterised by a cytosine to thymine transition, rs2419621 is located 12 nucleotides upstream of the second transcription start site of ACSL5. The presence of the [T] allele produces a new cis-regulating E-box site (DNA binding sites for E-proteins and myogenic regulatory factors such as MyoD) at the promoter region of ACSL5 in addition to the two wildtype E-box elements [9]. The presence of this extra E-box, has been shown to increase the amount of MyoD recruited to the ACSL5 promoter in vitro and to increase the expression of the downstream gene [9]. Furthermore, a 2.2-fold increase of ACSL5 transcript level was observed in skeletal muscle biopsies from individuals that are homozygous for the rs2419621 [T] allele when compared to homozygous wildtype individuals [8].

The objective of the present study was (1) to validate the influence of rs2419621 ACSL5 polymorphism on an independent population of women with obesity (2) To study the influence of rs2419621 on weight loss and body composition changes in response to lifestyle interventions on women with overweight and obesity. This article reports an increased response in women with overweight and obesity carrying the ACSL5 rs2419621 [T] allele to lifestyle interventions in comparison to non-carriers.

## Methods

### Subjects

Women categorized as being overweight or obese (*n* = 174) who participated in two hypocaloric lifestyle intervention studies Montréal Ottawa New Emerging Team study (MONET study) and Complication Associated with Obesity study (CAO study) were examined.

### Ethics, consent and permissions

All participants provided informed consent to engage in the lifestyle intervention as well as to the genetics components of these studies. Both cohort studies were approved by Université de Montréal ethics committee with agreement to the Declaration of Helsinki.

### MONET and CAO intervention studies

The MONET study (*n* = 137) and the CAO study (*n* = 37) recruited women between 2003 and 2007. The MONET study included postmenopausal women with overweight or obesity, while the CAO study population was composed of 35 women with obesity and two women with overweight. Both the MONET and the CAO cohorts and lifestyle intervention have been previously described and share major similarities [10]. Briefly, women were eligible to participate if they met the required criteria including: (1) Body mass index (BMI) ≥27 kg/m^2^, (2) menstruation ceased >1 year and plasma follicle-stimulating hormone levels ≥30 U l^−1^, (3) non-diabetic, (4) non-smokers, (5) no hormone replacement therapy, and (6) <2 h/week of structured exercise. The aim of MONET and CAO studies lifestyle intervention was a 10 % body weight reduction over 6 months. Caloric restriction targets for both studies were determined by subtracting 500 to 800 kcal from participants’ daily energy needs. The daily energy needs were calculated by multiplying resting metabolic rate of each participant (determined by indirect calorimetry [11]) to a physical activity factor of 1.4 as women were not exercising regularly at the onset of the trial. Dietary prescriptions ranged from 1100 to 1800 kcal/day. The diet macronutrient composition was 55, 30, and 15 % of energy intake from carbohydrates, fat, and protein, respectively [11]. A third of the MONET participants were enrolled in a resistance-training program whereas the CAO participants were not enrolled in resistance training. Subjects from both MONET and CAO studies were combined for statistical analyses after verifying that there were no significant differences between participants’ weight loss and body composition changes in response to the different lifestyle interventions.

### Studied variables

Analyses of these variables have been previously described [10]. Briefly, body weight measurement was conducted using a calibrated balance (Balance Industrielle Montréal, Québec, Canada) while other body composition variables, including fat mass and lean mass, were measured by Dual-energy X-ray absorptiometry (DXA) scans using the LUNAR Prodigy system (software version 6.10.019; General Electric Lunar Corporation, Madison, WI, USA). Height was measured using a wall stadiometer (Perspective Enterprises, Portage, MI, USA) while BMI was calculated as body weight (kg)/height(m)^2^. Visceral fat and thigh muscle attenuation were measured by computed tomography scan (General Electric Medical Systems, Milwaukee, WI, USA). Adjusting variables were measured using the COBAS INTEGRA 400 analyzer (Roche Diagnostic, Montréal Canada) for high-density lipoprotein cholesterol (HDL-C), triglycerides and glucose. The Friedwald formula was utilised to calculate cholesterol (LDL-C) concentration. C-reactive protein (CRP), haptoglobin, transferrin and orosomucoid levels were measured by immunonephelometry on an IMMAGE analyzer (Beckman Coulter, Villepinte, France).

### Isolation of DNA and rs2419621 genotyping using TaqMan MGB probe-based assay chemistry

Isolation of genomic DNA from the blood samples of individuals who participated in both MONET and CAO studies, was performed using a Qiagen Flexigene DNA kit. The ACSL5 rs2419621 SNP (CC, CT, TT) was genotyped using the TaqMan MGB probe-based assay chemistry (Life Technologies- Applied Biosystems). The PCR cycle (Biorad’s CFX-96 Real Time PCR) was as follows: enzyme activation at 95 °C for 10 min., denaturation at 92 °C for 15 s., 60 °C for 1 min-annealing/extension followed by 39 more repeats from the denaturation step. A positive control was used in order to ensure reliability of results. Biorad’s CFX96 real-time system was used for allelic discrimination while analysis of the data from end point fluorescence measurements were used to determine genotypes. Random samples were sequenced (Applied Biosystem) in order to confirm the genotype.

### Statistical analysis

Departure from Hardy-Weinberg proportions was tested on combined MONET and CAO participants using a Pearson’s chi-squared test.

Multiple linear regression analyses were conducted on subjects whose blood samples and biological parameter measurements (for dependent and adjusting variables) were available. Multiple linear regression analyses were conducted on: 1) All MONET and CAO participants and, 2) MONET and CAO women with obesity only (BMI ≥30).

Percentage changes in variables following intervention were calculated by subtracting post-intervention from baseline values divided by baseline values multiplied by 100. For this study we only had access to the pre and post-intervention values. Variables studied included BMI, fat mass, lean mass, visceral fat and thigh muscle attenuation. Both the absolute values and percentage values were studied for fat mass and lean mass [percent fat mass = (fat mass / total body mass) x 100,  with total body mass = fat mass + lean mass; percent lean mass = (lean mass / total body mass) x 100].

Regression analyses on the data sets for BMI, fat mass, lean mass, visceral fat and thigh muscle attenuation were conducted. Best subset regression analysis was conducted to determine which covariables should be included and adjusted for in the multiple linear regression analysis. Specifically we considered the following independent variables 1) based on their causative effect on weight loss: age, baseline weight, height, changes in transferrin, glucose, CRP, orosomucoid and haptoglobin 2) based on their correlation with weight loss: HDL-C, LDL-C, triglyceride [8]. The inclusion of a variable that is correlated with the response can reduce the error variance and increase the power of the regression analysis. The selection criteria to determine the best subset of independent variables was based on the adjusted R-squared and Mallows Cp. Specifically, we conducted a best subset regression analysis on data from populations consisting of women with overweight or obesity (Table 1) and women with obesity (Table 2). However prior to conducting any detailed statistical analyses, any extreme outliers found within the data sets of dependent and independent variables (if present) were excluded. Extreme outliers were determined based on observed values with a large standardized residual and large leverage. Specifically, one value from haptoglobin, transferrin and CRP was excluded, while two values were excluded for orosomucoid.

**Table 1:** Best subset regression analysis conducted on MONET and CAO women with overweight or obesity

Models of dependent variable studied	R-Sq (%)	R-Sq (%) (adj)	Covariables associated with mentioned R value
Change in BMI	13.1	8.5	Height, change in glucose, change in CRP, change in transferrin and change in triglyceride
Change in Lean Mass	17.8	12.7	Height, initial weight, change in HDL cholesterol, change in LDL cholesterol, change in triglyceride, change in glucose
Change in Fat Mass	21.3	16.4	Initial weight, change in glucose, change in CRP, change in transferrin, change in LDL cholesterol, change in triglyceride
Change in % Fat mass	28.7	23.6	Initial weight, change in HDL cholesterol, change in glucose, change in CRP, change in transferrin, change in LDL cholesterol, change in triglyceride
Change in % Lean mass	25.0	20.3	Initial weight, change in HDL cholesterol, change in orosomucoid, change in CRP, change in triglyceride, change in transferrin
Change in Visceral fat	15.9	11.4	Age, height, change in CRP, change in LDL cholesterol, change in triglyceride
Change in Muscle attenuation	14.0	11.0	Height, change in HDL, change in orosomucoid

* Results used to determine covariables for the multiple linear regression analysis. Data sets that were used for dependent variables were data from the MONET and CAO studies

**Table 2:** Best subset regression analysis conducted on MONET and CAO women with obesity

Dependent variable studied	R-Sq (%)	R-Sq (%) (adj)	Covariables associated with mentioned R value
Change in BMI	13.2	8.8	Change in transferrin, change in triglyceride and change in orosomucoid
Change in Lean Mass	13.8	8.2	Initial weight, change in HDL cholesterol, change in triglyceride, change in transferrin and change in CRP
Change in Fat Mass	21.6	15.4	Height, initial weight, change in CRP, change in transferrin, change in triglyceride
Change in % Fat mass	25.4	19.5	Height, initial weight, change in CRP, change in transferrin, change in triglyceride
Change in % Lean mass	25.7	18.7	Height, initial weight, change in HDL cholesterol, change in CRP, Change in transferrin, change in triglyceride
Change in Visceral fat	21.0	15.9	Age, initial weight, change in CRP and change in triglyceride
Change in Muscle attenuation	17.6	12.3	Change in HDL cholesterol, change in transferrin, change in orosomucoid and change in haptoglobin

* Results used to determine covariables for the multiple linear regression analysis. Data sets that were used for dependent variables were data from the MONET and CAO studies

In order to determine whether MONET & CAO populations could be pooled together for statistical analysis, unpaired t-tests were conducted to test for study effect. Both pre-intervention and post-intervention parameter values were compared between lifestyle intervention studies. Multiple linear regression analysis was also conducted in order to observe study effect through the use of multiple dummy variables on data for women with overweight or obesity combined. Studies were conducted comparing CC carriers from the MONET study to CC carriers from the CAO study. Specifically independent variables included three dummy variables representing the subgroups (CAO-CC carriers, CAO-CT/TT carriers and MONET CT/TT carriers) with the exception of CC carriers from the MONET study and the covariables that were determined to be appropriate for adjustment. Covariables were determined through best subset regression models for each dependent variable (Table 1). Similar analyses were conducted comparing CT/TT carriers from the MONET study and CT/TT carriers from the CAO study. Analysis was also conducted on women with obesity alone using the appropriate covariables. Covariables were determined by best subset regression (Table 2).

A multiple linear regression analysis was used to evaluate the association between ACSL5 rs2419621 genotype and changes in anthropometric and metabolic characteristics following the lifestyle intervention. A dominant genetic model (CT/TT vs. CC) was utilized due to the lack of individuals homozygous for the [T] allele. The multiple linear regression models included the genotypes as an independent variable. The multiple linear regression models were conducted on data sets of dependent variables. Covariables that were included were only specific to the best subset regression conducted for each dependent variable (Tables 1 and 2). *P*-values <0.05 were considered statistically significant. Data analysis was performed with Minitab software 17.

## Results

### Comparison of pre- and post-intervention anthropometric variables' values in each studied cohort and test for intervention effects

Pre- and post-intervention anthropometric variables from the MONET and CAO cohorts were compared using unpaired t-tests (Table 3). No significant differences in age, height, percent fat and lean mass were found between the two cohorts pre-intervention. Furthermore, no significant differences were observed for pre- and post-intervention absolute values for lean mass, visceral fat, and thigh muscle attenuation. However, significant differences were observed between pre- and post-intervention values for BMI and absolute value of fat mass between the two cohorts, as well as post-intervention values for percent fat mass and percent lean mass.

**Table 3:** Pre- and post-intervention anthropometric variable values from MONET and CAO women with overweight or obesity

Biological factor observed	MONET study			CAO study			*P*-value
	Mean	Std. Dev.	Size (*n*)	Mean	Std. Dev	Size (*n*)	
Age (yrs)	57.9	4.87	106	56.5	4.11	29	0.155
Height (m)	1.61	0.06	106	1.63	0.08	29	0.068
Pre-Intervention							
BMI (kg/m^2^)	32.4	4.74	106	34.3	3.56	29	0.041
Lean Mass (kg)	42.8	6.66	106	45.6	7.44	29	0.054
Fat Mass (kg)	38.5	9.57	106	43.6	9.21	29	0.011
Lean Mass (%)	51.5	4.59	106	49.8	3.62	29	0.072
Fat Mass (%)	45.6	4.70	106	47.2	3.73	29	0.084
Visceral Fat (cm^2^)	185	54.7	105	200	56.5	29	0.197
Thigh Muscle Attenuation (HU)	48.8	3.34	106	48.2	3.04	29	0.326
Post-Intervention							
BMI (kg/m^2^)	30.3	4.76	105	32.3	3.69	29	0.042
Lean Mass (kg)	41.9	5.70	104	43.6	6.56	29	0.168
Fat Mass (kg)	33.9	10.1	104	40.1	9.51	29	0.004
Lean Mass (%)	54.2	5.36	104	50.9	4.56	29	0.003
Fat Mass (%)	42.6	5.52	104	45.9	4.76	29	0.004
Visceral Fat (cm^2^)	162	57.7	102	177	51.3	29	0.215
Thigh Muscle Attenuation (HU)	48.6	3.56	105	48.1	3.27	29	0.542

* Results are from unpaired t-tests. Test was conducted on data from all subjects whose blood samples were available for study

When analysing study effects through the use of multiple linear regression analysis using a general linear model, no significant difference was observed between the changes in variables following lifestyle intervention, between CC carriers in MONET and CAO studies. The same results were observed between CT/TT carriers in MONET and CAO studies, illustrating no study effect between the two cohorts of interest (Tables 4 and 5).

**Table 4:** Regression analysis comparing CC carriers of MONET to CAO in women with overweight or obesity

Multiple regression model	Dependent variable	Number of subjects (*n*)	Parameter estimate	Standard error	Variable *p*-value	R^2^	R^2^ (adjusted)	Model *p*-value
1	∆ BMI	123	0.15	1.55	0.923	0.130	0.069	0.038
2	∆ Lean Mass	124	−2.46	1.51	0.105	0.212	0.150	0.001
3	∆ Fat Mass	121	4.73	3.04	0.123	0.233	0.170	<0.001
4	∆ % Lean Mass	126	−2.98	1.72	0.085	0.268	0.212	<0.001
5	∆ % Fat Mass	121	3.98	2.04	0.053	0.315	0.252	<0.001
6	∆ Visceral Fat	124	2.40	5.41	0.658	0.155	0.096	0.011
7	∆ Muscle attenuation	128	0.10	1.02	0.921	0.134	0.091	0.007

* Multiple linear regression analysis was conducted. Independent variables included three dummy variables (CAO CC Carriers, CAO CT/TT carriers and MONET CT/TT carriers) with the exception of MONET CC carriers while adjusting for covariables determined by best subset multiple linear regression, independent for each model (Table 1). The following results illustrate parameter estimates, standard error, *p*-values corresponding to CAO CC carriers independent variable

**Table 5:** Regression analysis comparing CT/TT carriers of MONET to CAO in women with overweight or obesity

Multiple regression model	Dependent variable	Number of subjects (*n*)	Parameter estimate	Standard error	Variable *p*-value	R^2^	R^2^ (adjusted)	Model *p*-value
1	∆ BMI	123	0.36	1.50	0.812	0.130	0.069	0.038
2	∆ Lean Mass	124	−0.35	1.39	0.802	0.212	0.150	0.001
3	∆ Fat Mass	121	2.15	2.92	0.462	0.233	0.170	<0.001
4	∆ % Lean Mass	126	−1.10	1.68	0.513	0.268	0.212	<0.001
5	∆ % Fat Mass	121	1.73	1.89	0.361	0.315	0.252	<0.001
6	∆ Visceral Fat	124	1.28	4.97	0.798	0.155	0.096	0.011
7	∆ Muscle attenuation	128	0.58	0.95	0.543	0.134	0.091	0.007

* Multiple linear regression analysis was conducted. Independent variables included three dummy variables to identify the polymorphism group (CC CAO, CC MONET and CT/TT CAO) with the exception of CT/TT MONET while adjusting for covariables determined by best subset multiple linear regression, independent for each model (Table 1). Following results illustrate parameter estimates, standard error, *p*-values corresponding to CT/TT CAO independent variable

### Association analysis between ACSL5 rs2419621 genotype and changes in anthropometric variables following the lifestyle intervention

The ACSL5 rs2419621 genotype frequencies were in Hardy-Weinberg equilibrium (HWE). Data sets were analyzed using multiple linear regression analysis adjusted for confounding variables specific to each dependent variable, to determine the association between rs2419621 genotype and changes in anthropometric variables following the lifestyle intervention (Table 6). [T] allele carriers showed a statistically significant greater decrease in their visceral fat as well as their absolute and percent fat mass values and a statistically significant greater increase in their percent lean mass in comparison to non-carriers following the interventions.

**Table 6:** Regression analysis studying lifestyle intervention effect in CT/TT vs CC women with overweight or obesity

Multiple regression model	Dependent variable	Number of subjects (*n*)	Parameter estimate	Standard error	Variable *p*-value	r^2^ model	r^2^ adjusted model	Model *p*-value
1	∆ BMI	123	−1.36	0.87	0.121	0.130	0.085	0.012
2	∆ Lean Mass	124	0.93	0.81	0.254	0.193	0.145	0.001
3	∆ Fat Mass	121	−3.82	1.66	0.023	0.213	0.164	<0.001
4	∆ % Lean Mass	126	2.33	0.94	0.014	0.248	0.203	<0.001
5	∆ % Fat Mass	121	−2.99	1.08	0.007	0.287	0.236	<0.001
6	∆ Visceral fat	124	−5.74	2.87	0.048	0.153	0.110	0.003
7	∆ Muscle attenuation	128	0.97	0.56	0.085	0.131	0.103	0.002

* Multiple linear regression analysis was conducted. Parameter estimate of independent variable = Xt was studied. Physiological factors (dependent variables) were studied while adjusting for confounding factors independent for each model (Table 1). Analysis was conducted on MONET and CAO studies combined

### Comparison of pre- and post-intervention anthropometric variables' values in women with obesity from each cohort studied and test for lifestyle intervention effect

The pre- and post-intervention anthropometric variables' values from the MONET and CAO cohorts consisting of only women with obesity were compared using unpaired t-tests (Table 7). No significant differences were observed between the different cohorts’ pre- and post-intervention values for any of the biological dependent variables studied.

**Table 7:** Pre- and post-intervention anthropometric variable values from MONET and CAO studies in women with obesity

Biological factor observed	MONET study			CAO study			*P*-value
	Mean	Std. Dev.	Size (*n*)	Mean	Std. Dev	Size (*n*)	
Age (yrs)	58.1	4.91	62	56.7	4.18	27	0.230
Height (m)	1.61	0.06	62	1.63	0.08	27	0.189
Pre-Intervention							
BMI (kg/m^2^)	35.3	4.04	62	34.7	3.37	27	0.489
Lean Mass (kg)	45.5	6.82	62	46.1	7.38	27	0.693
Fat Mass (kg)	43.9	8.64	62	44.2	9.10	27	0.898
Lean Mass (%)	49.6	4.43	62	49.7	3.71	27	0.888
Fat Mass (%)	47.6	4.48	62	47.3	3.82	27	0.753
Visceral Fat (cm^2^)	207	51.9	61	206	52.8	27	0.927
Muscle Attenuation (HU)	48.5	3.70	62	48.6	2.20	27	0.833
Post-Intervention							
BMI (kg/m^2^)	33.0	4.42	61	32.8	3.24	27	0.813
Lean Mass (kg)	44.1	5.69	60	44.1	6.58	27	0.998
Fat Mass (kg)	39.1	9.90	60	41.1	8.78	27	0.361
Lean Mass (%)	51.9	5.28	60	50.4	3.99	27	0.184
Fat Mass (%)	45.1	5.41	60	46.5	4.19	27	0.254
Visceral Fat (cm^2^)	182	58.5	60	182	47.4	27	0.981
Muscle Attenuation (HU)	48.2	4.09	61	48.6	1.89	27	0.661

* Results are from unpaired t-tests. Test was conducted on data from all subjects whose blood samples were available for study

A multiple linear regression analysis using a general linear model was also utilised to test whether body composition parameter changes were influenced by lifestyle interventions. No significant difference was observed between the changes in outcome variables (specifically change in BMI, absolute and percent fat mass, absolute and percent lean mass, visceral fat and muscle attenuation) following the lifestyle intervention, between CC carriers with obesity in MONET and CC carriers with obesity in CAO cohorts. Similar results were observed when studying CT/TT carriers between the two cohorts illustrating no significant difference between both study lifestyle interventions (data not shown).

### Association analysis between ACSL5 rs2419621 genotype and changes in anthropometric variables following the lifestyle intervention in MONET and CAO women with obesity

Dependent variable data sets were analyzed using multiple linear regression analysis adjusted for co-variates, to determine the association between rs2419621 genotype and changes in anthropometric variables following the lifestyle intervention in women with obesity (Table 8). A statistically significant greater decrease in BMI, absolute and percent of fat mass values, as well as visceral fat was noticed following the lifestyle intervention in women with obesity carrying the [T] allele vs. non-carriers. A statistically significant greater increase in percent lean mass values was observed in [T] allele carriers vs. non-carriers women with obesity following the lifestyle intervention.

**Table 8:** Regression analysis studying lifestyle intervention effect in CT/TT vs CC women with obesity

Multiple regression model	Dependent variable	Number of subjects (*n*)	Parameter estimate	Standard error	Variable *p*-value	r^2^ model	r^2^ adjusted model	Model *p*-value
1	∆ BMI	87	−2.14	1.05	0.045	0.129	0.087	0.022
2	∆ Lean Mass	85	0.49	1.07	0.645	0.151	0.086	0.041
3	∆ Fat Mass	85	−5.13	1.97	0.011	0.212	0.151	0.004
4	∆ % Lean Mass	85	3.12	1.19	0.010	0.257	0.189	0.001
5	∆ % Fat Mass	85	−3.30	1.30	0.013	0.252	0.194	0.001
6	∆ Visceral fat	85	−9.53	3.47	0.007	0.214	0.164	0.002
7	∆ Muscle attenuation	87	1.20	0.72	0.098	0.177	0.126	0.007

* Multiple linear regression analysis was conducted. Parameter estimate of independent variable = Xt was studied. Physiological factors (dependent variables) were studied while adjusting for confounding factors independent for each model (Table 2). Analysis was conducted on MONET and CAO studies combined

## Discussion

In 2014, according to the World Health Organization (WHO), obesity was shown to be present in about 13 % of the world’s adult population, while 39 % of adults 18+ were overweight, with 40 % of women being overweight [12]. The objective of this study was to evaluate the influence of ACSL5 rs2419621 genotype on the changes in body composition parameters in response to lifestyle intervention in women with overweight or obesity. In order to do so, the body composition changes in women participating in two lifestyle weight-reducing interventions, the MONET (diet and/or resistance training) and CAO (diet) studies were analyzed according to their genotype.

Prior to conducting analysis to determine the influence of rs2419621 genotype, lifestyle intervention-related changes in body composition parameter differences were compared between the two cohorts in order to determine whether they could be combined for statistical analyses. The MONET and CAO study population and designs shared a lot of similarities [10]. Specifically, both studies used the same hypocaloric diet interventions. Both studies also included non-diabetic postmenopausal women, with the MONET study including women with overweight or obesity, while CAO focused on women with obesity. A portion (one third) of the MONET study participants were assigned to resistance training, in addition to the diet intervention. Furthermore, criteria for exclusion were similar between studies. Significant differences were observed between pre and post intervention BMI and absolute fat mass values between the two cohorts, as well as for percentage of fat mass and percentage of lean mass in post intervention. These observations were expected as CAO cohort was nearly exclusively composed of women with obesity. No significant differences in age, height, pre percentage of fat/lean mass, pre and post values for visceral fat, absolute lean mass and thigh muscle attenuation were identified. The significant differences seen in pre and post intervention values between the cohorts for various outcome variables studied when women with overweight or obesity were pooled together, were abolished once the population was narrowed down to women with obesity only. This indicates that rather than observing a study effect, significant differences observed in the pre and post-intervention values between MONET and CAO cohorts consisting of both women with overweight or obesity, was a result of difference in BMI between the two cohorts, with a much larger proportion of women being overweight in the MONET cohort.

Association analyses confirmed that women with overweight or obesity carrying the [T] allele were more responsive to the MONET and CAO lifestyle interventions in comparison to non-carriers. More specifically, carriers of the [T] allele had a greater decrease in fat mass, while also displaying a greater increase in lean mass in comparison to non-carriers. [T] allele carriers also displayed a greater decrease in their visceral fat in comparison to non-carriers. Furthermore, when considering just women with obesity, in addition to observing similar results as the study with individuals with overweight and obesity, [T] allele carriers also illustrated a greater decrease in their BMI in comparison to CC individuals.

The results of the present study demonstrate a greater decrease in visceral fat in carriers of the ACSL5 rs2419621 [T] allele, in comparison to non-carriers when subjected to lifestyle intervention aimed at weight reduction. Excess accumulation of visceral fat, characterised as fat packed between inner organs, is associated with an increased risk of metabolic syndrome [13]. Various proposed mechanisms, such as the “portal theory” have been suggested to explain the relationship between visceral adiposity and common cardiometabolic diseases. Specifically, the “portal theory” states a rise in lipolytic activity within visceral adipocytes, contributing to an increased delivery of metabolic by-products such as free fatty acids into the liver, eventually resulting in insulin resistance [14, 15]. Furthermore, elevated serum leptin levels are correlated with subcutaneous fat but not visceral fat deposition [16]. This decrease in serum leptin levels observed with omental fat tissue deposition contributes to a decreased regulation of appetite and impacts energy balance regulation [16].

Individuals with the rs2419621 [T] allele appeared to have a greater decrease in fat mass and a greater increase in their lean mass following the intervention, in comparison to non-carriers. While fat mass encompasses the overall fat composition of an individual, lean mass represents non-fat containing tissue mass including bone connective tissue, skin, organs and the muscle mass of an individual. Thus carriers of the [T] allele have a greater loss in fat and increase in lean body mass, in comparison to non-carriers.

Our results obtained from studying the effects of the rs2419621 polymorphism on subjects who participated in the MONET and CAO lifestyle intervention studies, validate our previous results from the 900-cal/day meal replacement study [8]. While our previous work focused on studying the effects of rs2419621 [T] allele carriers on rate of weight change, the present paper has shown the effects of the polymorphism on changes in body composition indices. Furthermore, the results from our current study illustrate that the rs2419621 polymorphism has a strong effect on subjects’ response to diet/ exercise intervention, resulting in moderate weight and fat loss, as statistically significant findings were observed even with interventions that did not have a strict meal replacement plan implemented.

At this point, the molecular mechanism by which the rs2419621 [T] allele exerts its effect is still unknown. However, the [T] allele has been shown to be associated with increased ACSL5 expression in rectus femoris muscle [8]. Furthermore, we showed, in vitro, that the [T] allele generates a cis-regulatory E-box element recognized by MyoD, a myogenic regulatory factor [9]. The [T] allele promotes MyoD-dependent activation of the ACSL5 promoter, suggesting a direct link between level of expression of ACSL5 and rs2419621 genotype [9]. Since ACSL5 is known to be mitochondrially localized, [T] allele carriers might present higher fat oxidation levels due to increased level of ACSL5.

## Conclusion

Based on the observed statistical analyses, carriers of the ACSL5 rs2419621 [T] allele have been shown to be more responsive to the MONET and CAO lifestyle interventions in comparison to non-carriers. The strong association observed between rs2419621 [T] allele and response to diet/exercise intervention, has led to the hypothesis of individuals with the [T] allele having increased fat oxidation in comparison to individuals with the wild type. Our study confirms and provides additional insights on the influence of the rs2419621 polymorphism in response to lifestyle interventions as it is an expansion of the previous work from Adamo et al. 2007 [8]. However, it should be noted that population stratification was not controlled for in our study. As our current study focused on the effect of rs2419621 on postmenopausal women, future directions include replicating these findings in a large male population or male and female population. Furthermore future work with in vitro/ in vivo models studying the regulation of fatty acid β-oxidation by the rs2419621 [T] polymorphism are needed. Such genetic association studies can aid in designing individualized treatments/interventions for weight loss catered towards an individual’s genotype specifically by modifying both diet and exercise.
